# Hospital Meetings

**Published:** 1901-07-06

**Authors:** 


### HOSPITAL MEETINGS.

### THE HOSPITAL FOR WOMEN.

Lord Shaftesbury, the president, took the chair at the fifty-eighth annual meeting of the Hospital for Women, Soho Square, on the 20th instant. The secretary read the report of the Committee of Management, which opened with the announcement that Her Majesty Queen Alexandra had consented to continue her patronage of the hospital. During the year 1900 the record number of 738 in-patients were admitted, this being an increase of 32 over the previous year. The total number of in-patients under treatment during the year was 780, with a death-rate of 2·05 per cent. Of the 16 deaths 4 occurred without any operative interference having been undertaken. The average number of beds occupied daily throughout the year was 49, the average stay of each patient being 24 days. The new out-patients numbered 4,490, with 20,880 attendances—a decrease of 15 new cases but an increase of 903 attendances. During August the out-patient department was closed for 3 weeks; 67 patients were sent to Convalescent Homes through the aid of the Reardon and Sturge Samaritan Funds. The income for the year was £4,635, and the expenditure £6,226—a deficit of £1,590—which, added to that of the previous year made the sum of £5,445 owing on December 31st, 1900. The vacancy on the board occasioned by the resignation of Mr. Abel Smith, M.P., was filled by the appointment of Dr. James Oliver. The committee also reported the resignation of Dr. Holland, one of the senior physicians, after twenty-six years' service, and expressed the hope that the Governors would appoint him a consulting physician to the hospital in recognition of his long service on behalf of the institution. Dr. Bedford Fenwick, senior physician in charge of out-patients, had been appointed physician in charge of in-patients, and the committee nominated Mr. Laurie H. McGavin for election as assistant-surgeon. In conclusion, the committee expressed regret that, owing to the want of adequate funds, their efforts to maintain the full number of beds in constant service throughout the year proved unsuccessful. All the beds (64)

however, were kept in use until September 1st, when it was found to be absolutely necessary to reduce their number in order to curtail expenditure. This course had not been found necessary for a period of twenty-three years, and the committee expressed the hope that it might be only a temporary arrangement, inasmuch as it caused much suffering to the many applicants for indoor treatment, by necessitating a delay in their admission to the wards of several weeks, when formerly, with all the beds available, a few days were sufficient.

In moving the adoption of the report the Chairman expressed regret that they were rather in a worse position than when he addressed them last year. It was an unfortunate state of affairs, and it was difficult to suggest a remedy. He thought there could be no doubt that the committee had acted wisely in curtailing the number of beds.

Mr. Victor Williamson, chairman of the Committee of Management seconded. He said he was afraid their position was really very serious. They were living at the rate of £1,600 a year over their income, and there was no doubt that that could not last. In reducing the number of beds from 64 to 39 they had adopted the course which prudence dictated and indeed necessitated. But there were then 52 cases waiting to be taken in, and of these 10 were marked "urgent" by the medical officers. This, of course, meant much suffering for the poor women involved. Some years back, when they had had several fat years, they spent £10,000 in bricks and mortar, but any who recalled the state of their building at that time would justify their action. Their nurses' quarters especially were a perfect scandal—they were not safe, and were not sanitary. No charge of extravagance could fairly be brought against them for pulling down the old place and making the hospital safe and clean. As to the future, they contemplated the idea of a dinner to raise funds, and it had been decided to call a special meeting to look ahead with regard to organising the matter for next year.

The retiring members of the committee were re-elected; further leave of absence was granted to Mr. Douglas Drew, the assistant surgeon, on service in South Africa; Dr. Holland was elected consulting physician, and Mr. Lawrie H. McGavin assistant surgeon; and the meeting was then made special for the purpose of alterations in the bye-laws providing that admission of out-patients should be by letters of recommendation after the first visit, or on payment of 1s. for each day's attendance.

Mr. Harvie said a similar plan had been adopted with success at the Chelsea Hospital for Women, about £400 a year being produced. With double the number of out-patients they might fairly hope to add at least £500 or £600 to their revenue.

The motion was agreed to, and a vote of thanks to the Chairman concluded the proceedings.

### HOSPITAL FOR EPILEPSY AND PARALYSIS.

The Bishop of Stepney presided at the thirty-fourth annual meeting of the Hospital for Epilepsy and Paralysis and other Diseases of the Nervous System, Regent's Park, on June 26th ultimo. The report and accounts for 1900 were presented by the secretary, and from these it appeared that the ordinary income was £1,068 (a decrease of £300) and the ordinary expenditure £1,444 (about £200 less than in 1899). The extraordinary income (legacies) was £5,389, and the extraordinary expenditure (expenses of incorporation) £42—£5,000 being transferred to the New Building Fund and a loan of £100 repaid, while the cash in hand was £93. The report went on to refer to the fact that since May, 1900, the institution had been incorporated, the step having been rendered necessary in connection with business arrangements involved by the acquisition of the site for the new building. The lease of Winterton House, so long occupied by the hospital, expired in 1900, but the site not being as yet actually required for building purposes, an extended occupation had unexpectedly become possible and it was hoped that temporary premises would not be found necessary. In July, however, the committee were compelled with great regret to arrange for the discharge of the in-patients and nurses. While regretting that the acquisition of a suitable freehold for the new building was found impossible, the Committee congratulated their supporters on having been able to acquire a lease for 99 years at a yearly rental of £165 of the site at 4 Maida Vale. The hospital would thus be established very near to their old premises, in a very eligible and accessible position, in a wide and wood-paved thoroughfare. The expenses of acquiring this site were by the kindness of Mr. Geo. Fisher, a member of the Committee, freed from all legal charges; but the sum of £1,878 appearing in the accounts of the new building fund included the premium and the fees and costs of the surveyor and solicitor to the trustees of the Harrow Estate.

The Committee gave most careful consideration to various plans submitted to them by Mr. Keith D. Young, F.R.I.B.A., who was chosen from among twelve gentlemen whose names and qualifications were before the Committee; and from his plans they selected one which, when fully carried out, would, they considered, ultimately result in a building providing far more effectually for the needs of the hospital than would have been possible under alternative schemes, though these might, perhaps, have been less costly. The adoption of this plan has, however, entailed a much larger expenditure on the first portion of the building than the Committee expected; for the lowest of the tenders received from the nine firms invited to compete was found to be over £20,000. The Committee having in hand, after the acquisition of the site, a sum of only £12,000, learned, with great regret, that their requirements so far exceeded the means at their disposal; but they decided that, rather than imperil the future efficiency and completeness of the building, they would adhere to the superior but more costly plan, and limit, to a very large extent, its immediate development, by entering into a contract for the erection of the structure, up to the level of the first floor only, with the option, however, of ordering further additions, if the funds placed at their disposal should justify that course.

It was for the friends of the hospital themselves to decide, by their contributions and their support, whether the work must for the time stop at this point, or whether, by supplying the £8,000 absolutely required before Michaelmas, they would encourage the committee to proceed with the execution of the complete contract of £20,000 for the first portion of the building. In the meantime, the work of erecting the new building was proceeding within the limitations indicated.

The committee recorded with regret the loss by death of two vice-presidents, the Marquis of Bute and the Earl of Darnley, elected in 1883 and 1884 respectively. The lamented death in May of Dr. Althaus, by which the hospital was deprived of its founder and the committee of their oldest member, was referred to in the last report. Since the previous March Dr. Althaus, finding himself unable to attend the committee meetings, had tendered his resignation, Dr. Turner being appointed in his place. Dr. Turner having resigned in June in consequence of duties undertaken at another hospital, Dr. Johnston was appointed to take charge of in-patients and was elected a member of the Committee of Management, to which also Messrs. J. Edmund Bentley and R. Martin Holland had been added. The circumstances relating to the occupancy of Winterton House and the probable necessity of temporary premises led to the closure of the out-patient department during September, and a diminution in the number of "attendances," 10,169 as compared with 11,259 in 1899, was no doubt attributable in some measure to this step. The figure, however, still exceeded that of any year earlier than 1896. In consequence of the interruption of that portion of the work since August the number of in-patients under treatment was only 60.

The CHAIRMAN, in moving the adoption of the Report, referred in sympathetic terms to the pathos of the suffering caused by epilepsy and paralysis with which he had been brought much into contact, and expressed great regret that the in-patient department should have had to be closed. It was most urgently necessary that the work so ably done there should not be crippled for any length of time. He could not but think it strange that a hospital which had done so much good work should have reached such a crisis. It was the more to be regretted, since many of the cases dealt with there were excluded from most of the general hospitals.

Canon BARKER seconded the motion, and while agreeing with the Bishop that it was most regrettable that there should be any interruption in the work, yet felt bound to congratulate the society on the success already attained in their efforts to obtain more adequate and suitable premises. They had secured a site and £12,000 towards the cost of the new building. It was a really fine site, in a beautiful neighbourhood; and when the building to be erected had materialised and become actual fact in the form represented in the very noble picture they had before them the contrast between it and their present cramped and unsuitable surroundings would be a very notable and advantageous one. They had occupied the present premises for some 34 years, and had done much good work there; and he was glad to think that after working for so many years and treating such an immense variety of diseases as the carefully tabulated medical statement showed they did in that humble place that they were now going to have so grand and splendid a building, and one so admirably adapted to their work.

The report was adopted, and Mr. PEARCE GOULD, one of the consulting surgeons, proposed the re-election of Dr. G. Ogilvie, Captain R. A. F. W. Ellis, and Mr. J. Ogle as members of the Managing Committee. Referring to the fact that the first-named gentleman was one of the senior medical officers, he said they had recently seen much evil arising from the neglect of the wise provision under which they at that hospital worked, that members of the medical staff should be joined with laymen on the Managing Committee, and under which they had always worked happily together. He emphasised the need for hospital treatment of diseases of the brain and nervous system, more especially on the surgical side, and reminded the meeting that it was at that institution that the first great impetus to surgical effort had been given when the first operation for tumour of the brain was carried out.

The motion was seconded by Mr. G. A. FISHER—a lay member of the committee, who also emphasised the harmony obtaining on the managing body—and agreed to, after which the meeting separated.

